# Research on remote sensing classification of fruit trees based on Sentinel-2 multi-temporal imageries

**DOI:** 10.1038/s41598-022-15414-0

**Published:** 2022-07-07

**Authors:** Xin-Xing Zhou, Yang-Yang Li, Yuan-Kai Luo, Ya-Wei Sun, Yi-Jun Su, Chang-Wei Tan, Ya-Ju Liu

**Affiliations:** 1 Xuzhou Institute of Agricultural Sciences in Jiangsu Xuhuai District, Xuzhou, 221131 Jiangsu China; 2 Jiangsu Key Laboratory of Crop Genetics and Physiology / Jiangsu Co-Innovation Center for Modern Production Technology of Grain Crops / Joint International Research Laboratory of Agriculture and Agri-Product Safety of the Ministry of Education of China, Yangzhou University, Yangzhou, 225009 Jiangsu China

**Keywords:** Environmental monitoring, Environmental impact

## Abstract

Accurately obtaining the spatial distribution information of fruit tree planting is of great significance to the development of fruit tree growth monitoring, disease and pest control, and yield estimation. In this study, the Sentenel-2 multispectral remote sensing imageries of different months during the growth period of the fruit trees were used as the data source, and single month vegetation indices, accumulated monthly vegetation indices (∑VIs), and difference vegetation indices between adjacent months (∆VIs) were constructed as input variables. Four conventional vegetation indices of NDVI, PSRI, GNDVI, and RVI and four improved vegetation indices of NDVIre1, NDVIre2, NDVIre3, and NDVIre4 based on the red-edge band were selected to construct a decision tree classification model combined with machine learning technology. Through the analysis of vegetation indices under different treatments and different months, combined with the attribute of Feature_importances_, the vegetation indices of different periods with high contribution were selected as input features, and the Max_depth values of the decision tree model were determined by the hyperparameter learning curve. The results have shown that when the Max_depth value of the decision tree model of the vegetation indices under the three treatments was 6, 8, and 8, the model classification was the best. The accuracy of the three vegetation index processing models on the training set were 0.8936, 0.9153, and 0.8887, and the accuracy on the test set were 0.8355, 0.7611, and 0.7940, respectively. This method could be applied to remote sensing classification of fruit trees in a large area, and could provide effective technical means for monitoring fruit tree planting areas with medium and high resolution remote sensing imageries.

## Introduction

Scientifically, quickly and accurately classifying the main producing areas of fruit trees is the basis for carrying out research on the growth of fruit trees, prevention of diseases and insect pests, and yield estimation, and is of great significance to agricultural production^1,2^. Traditional manual survey methods are inefficient and costly, and some subjective factors, such as statistical errors, inconsistent standards in various regions, and different measurement tools, will inevitably affect the accuracy of statistical surveys in the statistical process. Remote sensing technology has been widely used in the estimation of agricultural planting area and the optimization of planting area due to its advantages such as objectivity, timeliness and large-scale implementation, and at the same time, it has low economic investment and is not subject to geographical restrictions^3–5^.

At present, most researches on fruit trees have been concentrated in the fields of cultivation measures, occurrence of diseases and insect pests, and physiology and biochemistry. There were relatively less studies on the classification of planting areas using full-time sequence remote sensing imageries to analyze the spectrum of fruit trees and combined with machine learning technology, and the classified and extracted of crops were mainly concentrated in large-scale crops such as wheat, rice, maize, and etc.^6–8^. In the past several decades, a variety of remote sensing methods that use different types of remote sensing data to discriminate crop types have been developed^9^. This includes radio detection and ranging (RADAR) data, which is beneficial because it can penetrate clouds and thus solve the problem of cloud contamination. However, RADAR data has been shown to have issues with low resolutions, high levels of noise, and high cost^10,11^. Hyperspectral imagery has also been used for crop discrimination, but due to their prohibitively high cost, there were obstacles in actual production applications^12^. Limitations associated with RADAR and hyperspectral data led to the moderate resolution imaging spectroradiometer (MODIS) and advanced very high-resolution radiometer (AVHRR) being the most exploited datasets for crop type classifications^13,14^.

Time series remote sensing imageries have not only the spectral information of a single-phase imagery, but also a series of time information, which is of great significance in the extraction of crop distribution information. Compared with Landsat-8, MODIS and other data, Sentinel-2 satellite imagery has certain advantages in temporal and spatial resolution and spectral information^15,16^. Based on the excellent spatio-temporal and spectral information characteristics of Sentinel-2 satellite imageries, it effectively improved the classification accuracy of related crops in the identification of precise crop types in small agricultural areas^17^. Relevant studies have shown that multi-temporal Sentinel-2 data may significantly improve classification accuracy. The effective combination of high spatial–temporal resolution and rich spectral information provides an unprecedented rich data set from which crop types can be accurately distinguished^18^. It is generally believed that multi-temporal data is important for crop type classification. Choosing the most useful imageries from the time series for research usually yields more accurate results than using all available imageries. This was supported by some studies show that imageries obtained during peak growth are more useful than imageries obtained during low growth^19,20^. This was attributed to the observation that some crops might be spectrally similar or constitute high levels of soil interference due to a lack of growth during certain stages in the growing season^21,22^.

In summary, the current research on extracting crop planting distribution in a large area principally includes the following aspects. The first is the research on crop recognition ability with remote sensing under the conditions of different remote sensing imageries; the second is the comparative research on crop recognition ability and accuracy based on different classification methods; the third is the combination of multi-temporal imageries and multi-features to assist crop classification. Generally speaking, there have been more studies on the best method and the highest precision under the condition of fully guaranteeing the data source^23–25^. However, the remote sensing identification of crops has been mainly concentrated on the stable crops, and there were few related researches on the remote sensing identification of fruit trees. This study used multiple Sentinel-2 imagery spectral data from November 2020 to October 2021, took the Dashahe area of Jiangsu Province as the research area, combined machine learning technology to build a decision tree classification model, and conducted detailed classification of fruit tree categories in the fruit tree planting area. In this way, the visualized results of the spatial layout of fruit tree classification were obtained, which was expected to provide relevant basis for the application of medium and high-resolution remote sensing data for fruit tree surveys, and to provide support for fruit tree planting subsidies and production layout planning.

## Materials and methods

### Study region experimental design

**Figure 1:**
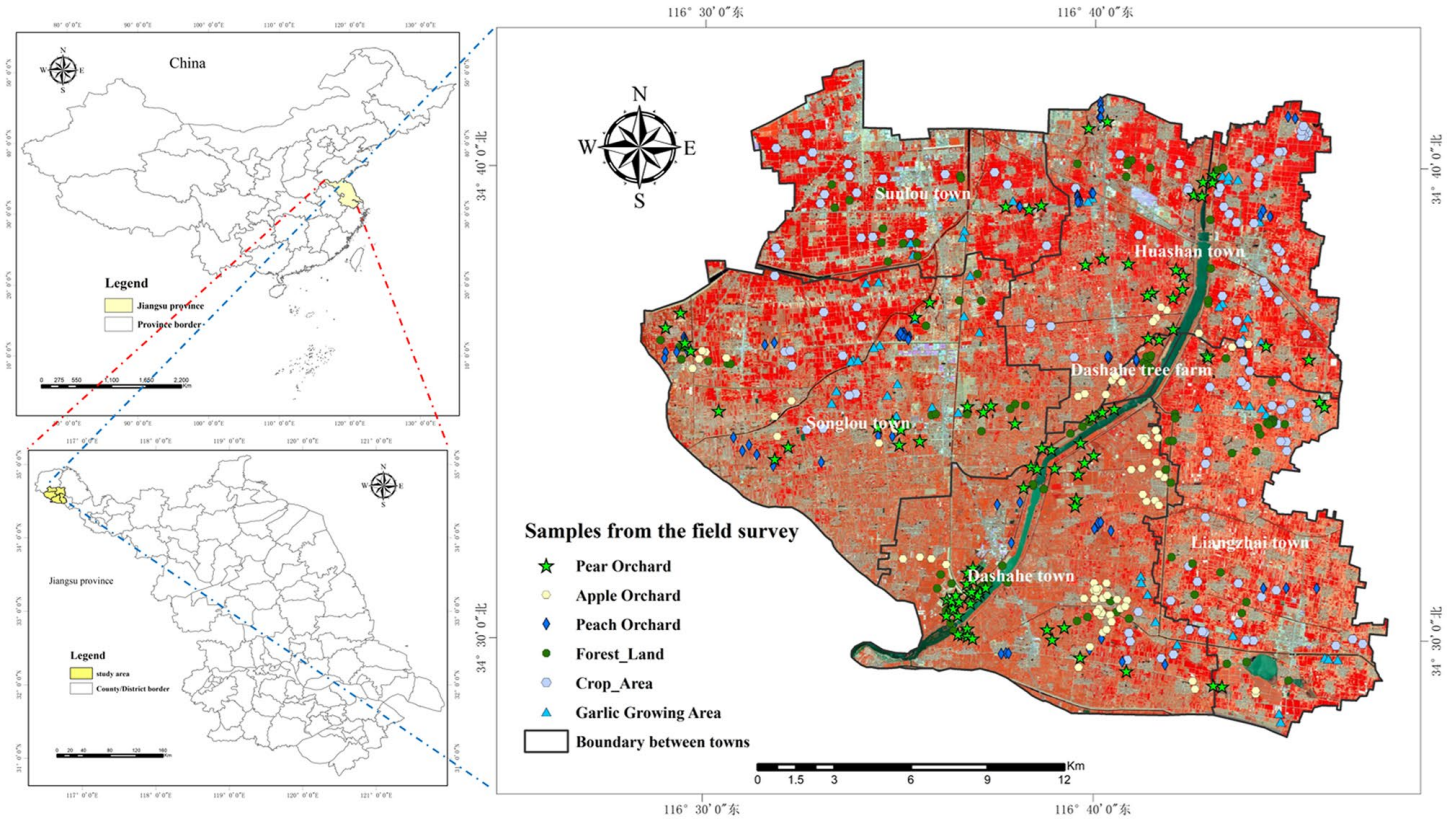
Location of study area. The picture on the left shows the map of China and the province map where the research area is located. The picture on the right is a false-color image of the study area of the Dasha River Basin, which includes the collection points of different features. Map created in ENVI5.3 and ArcMap 10.7 (ESRI Co., USA (https://envi.geoscene.cn/)/(https://www.esri.com/software/arcgis/arcgis-for-desktop)). Boundaries made with free vector data provided by National Catalogue Service for Geographic Information (https://www.webmap.cn/commres.do?method=dataDownload). The satellite imagery data source: Sentinel-2 (https://scihub.copernicus.eu/dhus/#/home).

As the principal fruit production area in northern part of Jiangsu was selected as the study region. The study was conducted in six towns in Feng city, Jiangsu Province (116°28′E-121°47′E, 34°28′N-34°42′N) in the 2020 and 2021 fruit tree growing seasons. The experiment was carried out around Sunlou Town, Huashan Town, Songlou Town, Dashahe Town, Liangzhai Town and Dashahe Forest Farm in the Dasha River Basin (Fig. 1). Fengxian County is located in a warm temperate semi-humid monsoon climate zone, with four distinct seasons, abundant sunshine, and an average annual temperature of about 15 °C. Its terrain is a yellow river flooded alluvial plain, high-pitched and flat. The rivers in the territory were originally natural rivers, criss-crossed, and the abandoned tributary of Yellow River was treated and introduced into the Yangtze River to form a belt-shaped reservoir in the Dasha River Basin. The Dasha River Basin has fertile land and abundant products, with a large area for planting crops, fruit trees, and horticultural vegetables. Based on the unique natural conditions, the fruit trees in the Dasha River Basin have been planted for decades, and the area is huge, so it has become a well-known fruit production base.

**Figure 2:**
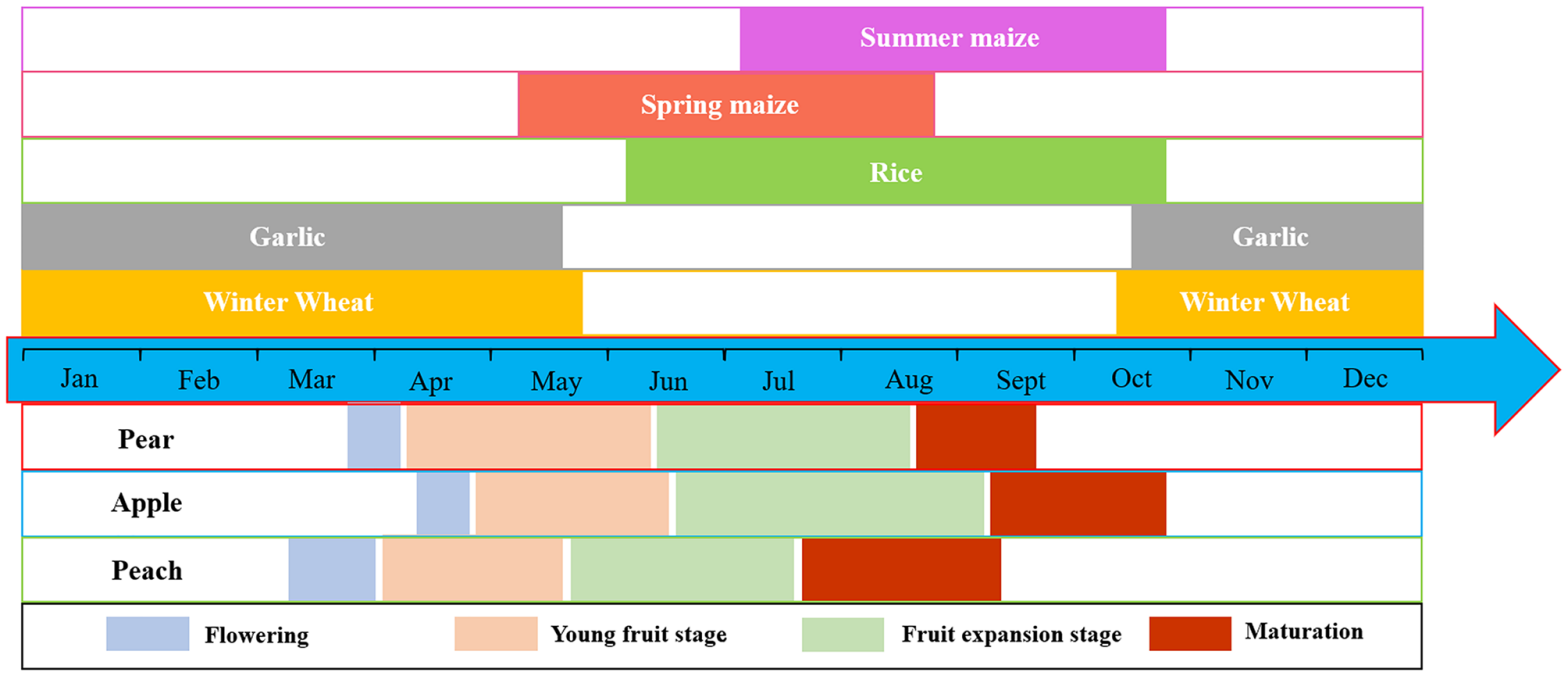
Growth cycle of main crops and fruit trees in the Dasha River Basin. The top is the growth period of the main field crops, and the bottom is the growth period of the main fruit trees. Due to the different cultivars and cultivation methods, the growth cycle of the same species in the study area is not completely consistent. This diagram reflects the approximate growth cycles of different crops and fruit trees.

The main field crops in the Dasha River Basin are wheat, corn and rice, as well as horticultural vegetable garlic and so on. The fruit trees in the study area are mainly pear trees and apple trees, and some peach trees are planted in the area too. All the fruit trees are dormant in winter. The trees germinate from mid-to-late February to early March, flower from March to April, then bear fruits one after another, and mature from July to November. Figure 2 shows the growth cycle of the main crops and fruit trees in the study area.

### Remote sensing imagery data

Sentinel-2 is the second satellite of the "Copernicus Project" in Europe, with two satellites in total. As a rising star, Sentinel series of data imageries have received more and more attention in research and production due to their high-quality temporal and spatial resolution and rich band information. Sentinel-2A/B products were downloaded through the data sharing website of the European Space Agency (ESA) (download URL: https://scihub.copernicus.eu/dhus/#/home). The products used in this study were all L2A level, i.e., they had undergone geometric correction and atmospheric correction processing, which effectively removed the influence of the atmosphere, clouds and the reflection of ground. At the same time, all bands were resampled to 10 m resolution in the SNAP software for fusion. From November 2020 to October 2021, a total of 12 imageries were selected for the experiment, and the imagery number was N0300-R032-T50SMD. This study selected four 10 m resolution bands and four 20 m resolution red-side bands. The range and resolution of each band are shown in Table 1.

**Table 1:** Band and resolution information of Sentinel-2 imagery in this research.

Sentinel-2 band	Central wavelength (um)	Resolution (m)
Band 2-Blue	490	10
Band 3-Green	560	10
Band 4-Red	665	10
Band 5-Vegetation Red Edge	705	20
Band 6-Vegetation Red Edge	740	20
Band 7-Vegetation Red Edge	783	20
Band 8-Near Infrared	842	10
Band 8A-Vegetation Red Edge	865	20

The vegetation indices are of important indicator to measure the growth of crops and distinguish the crop types. In the research of crop classification, normalized differential vegetation index (NDVI), ratio vegetation index (RVI), plant senescence reflectance index (PSRI) and green normalized differential vegetation index (GNDVI), four vegetation indices based on visible light and near-infrared wavebands, were widely used^26–29^. On the basis of calculating the existing conventional vegetation indices, the red edge bands of the Sentinel-2 imagery were introduced to replace the visible light band or the near-infrared bands of the NDVI, so as to calculate new vegetation indices. Normalized difference vegetation index red-edge1 (NDVIre1) used band 5 instead of the red band. Normalized difference vegetation index red-edge2 (NDVIre2), normalized difference vegetation index red-edge3 (NDVIre3) and normalized difference vegetation index red-edge4 (NDVIre4) used band 8A to replace the near-infrared band, and band 5, band 6 and band 7 respectively to replace the red band in NDVI^30–33^. The calculation method of the relevant vegetation index is shown in Table 2.

**Table 2:** Vegetation index calculation formula.

Vegetation index	English full name	Calculation formula
NDVI	Normalized Differential Vegetation Index	$\frac{Band8 - Band4}{{Band8 + Band4}}$
PSRI	Plant Senescence Reflectance Index	$\frac{Band4 - Band2}{{Band8}}$
GNDVI	Green Normalized Differential Vegetation Index	$\frac{Band8 - Band3}{{Band8 + Band3}}$
RVI	Ratio Vegetation Index	$\frac{Band8}{{Band4}}$
NDVIre1	Normalized Differential Vegetation Index red-edge1	$\frac{Band8 - Band5}{{Band8 + Band5}}$
NDVIre1	Normalized Differential Vegetation Index red-edge2	$\frac{Band8A - Band5}{{Band8A + Band5}}$
NDVIre1	Normalized Differential Vegetation Index red-edge3	$\frac{Band8A - Band6}{{Band8A + Band6}}$
NDVIre1	Normalized Differential Vegetation Index red-edge4	$\frac{Band8A - Band7}{{Band8A + Band7}}$

### Ground spectral data acquisition

Based on the multi-spectral reflectance imageries of multi-temporal Sentinel-2, through sample points of field survey (Fig. 1) and visual interpretation combined with Google imageries, 210 pear tree sample points, apple tree sample points, peach tree sample points, and forest land sample points were selected respectively in the study area for the composition of the training set and test set of the decision tree model, and 185 sample points of crop planting areas and 104 sample points of garlic planting areas were selected for the classification of field crop planting areas and other vegetation areas (Table 3). Among them, buildings and water bodies could be directly removed by supervision classification masks, so this study would not discuss them.

**Table 3:** The number of samples of different types.

Types of samples	Pear orchard	Apple orchard	Peach orchard	Forest land	wheat & corn rotation	wheat & rice rotation	Garlic growing area
Number of samples	210	210	210	210	138	47	104

### Vegetation indices processing and accuracy verification

Different types of vegetation often cannot show significant differences in the spectrum of the same period, so it is necessary to combine multi-temporal remote sensing data to make judgments. On this basis, the accumulation and subtraction of multi-temporal spectral data can fully excavate the multi-temporal data information, amplify the spectral information differences between vegetation types, and explore the performance of different spectral information processing. In order to explore the effect of vegetation indices on fruit tree classification under different treatments, this study used single month vegetation indices (SVIs), accumulated monthly vegetation indices (∑VIs), and difference vegetation indices between adjacent months (∆VIs) to analyze separately^34,35^. The SVIs was the vegetation index constructed from the spectral information extracted from the imagery corresponding to that month. The ∑VIs was the sum of the vegetation indices constructed from the spectral information extracted from the corresponding imageries of the month and all previous month (Eq. (1)). The ∆VIs was the difference between the vegetation indices constructed from the spectral information extracted from the corresponding imageries of the month and the previous month (Eq. (2)).

1 $$ \Sigma VI = \mathop \int \limits_{1}^{n} VI{\text{d}}t $$

2 $$ \Delta VI = VI_{n} - VI_{n - 1} $$

where n is the month corresponding to the vegetation index.

Classification accuracy is an important indicator used to measure the advantages and disadvantages of classification model. Common metrics include user accuracy, mapping accuracy, and Kappa coefficient^36,37^. In this paper, user accuracy, mapping accuracy, and Kappa coefficient were selected as accuracy evaluation indicators based on the confusion matrix calculation. The accuracy of the classification method was comprehensively evaluated as follows:

User accuracy of the $i$ th class of data ($P_{ui}$),

3 $$ P_{ui} = {\raise0.7ex\hbox{${p_{ii} }$} \!\mathord{\left/ {\vphantom {{p_{ii} } {p_{i + } }}}\right.\kern-\nulldelimiterspace} \!\lower0.7ex\hbox{${p_{i + } }$}} $$

Mapping accuracy of the $j$ th class of data ($P_{AJ}$),

4 $$ P_{AJ} = {\raise0.7ex\hbox{${p_{jj} }$} \!\mathord{\left/ {\vphantom {{p_{jj} } {p_{ + j} }}}\right.\kern-\nulldelimiterspace} \!\lower0.7ex\hbox{${p_{ + j} }$}} $$

Kappa coefficient

5 $$ Kappa = \frac{{P\mathop \sum \nolimits_{i = 1}^{n} p_{ii} - \Sigma_{{{\text{i}} = 1}}^{n} \left( {p_{i} + p_{ + j} } \right)}}{{P^{2} - \Sigma_{{{\text{i}} = 1}}^{n} \left( {p_{i} + p_{ + j} } \right)}} $$

where $p_{ii}$ represents the number of correct classifications of the sample type in the $i$ throw and $i$ th column of the confusion matrix, The $p_{jj}$ is the same. $P_{i + } = \Sigma_{{{\text{i}} = 1}}^{n} p_{ij}$ is the sum of the number of $i$ th class pixels in the classification result. $P_{ + j} = \Sigma_{{{\text{i}} = 1}}^{n} p_{ij}$ is the sum of the $j$ th class pixels in the measured data. P is the total number of samples, and n is the number of classes.

### Decision tree classification based on machine learning technology

The software tool used in this study for data processing and construction of decision tree models was Python2.7^38^. Among them, the Scikit-learn module, also written as Sklearn, is an open source machine learning toolkit based on the Python language. Through numerical computing modules such as NumPy, Pandas, SciPy and Matplotlib, efficient algorithm applications can be realized, and it covers almost all algorithms^39^. The decision tree model used in this article was also selected from the Sklearn machine learning module. Before starting the construction of the decision tree model, it is of great significance to determine the appropriate input features for the final classification result and the complexity of the model. Based on this, the Feature_importances_ attribute in the model could be combined to obtain the feature importance value, and unimportant features could be filtered out before model fitting, in order to obtain better stability and accuracy. At the same time, there was a very important parameter in the decision tree classification model as the Max_depth parameter which function was to limit the maximum depth of the tree. The best Max_depth value could make the decision tree model have better performance.

### Technical route

**Figure 3:**
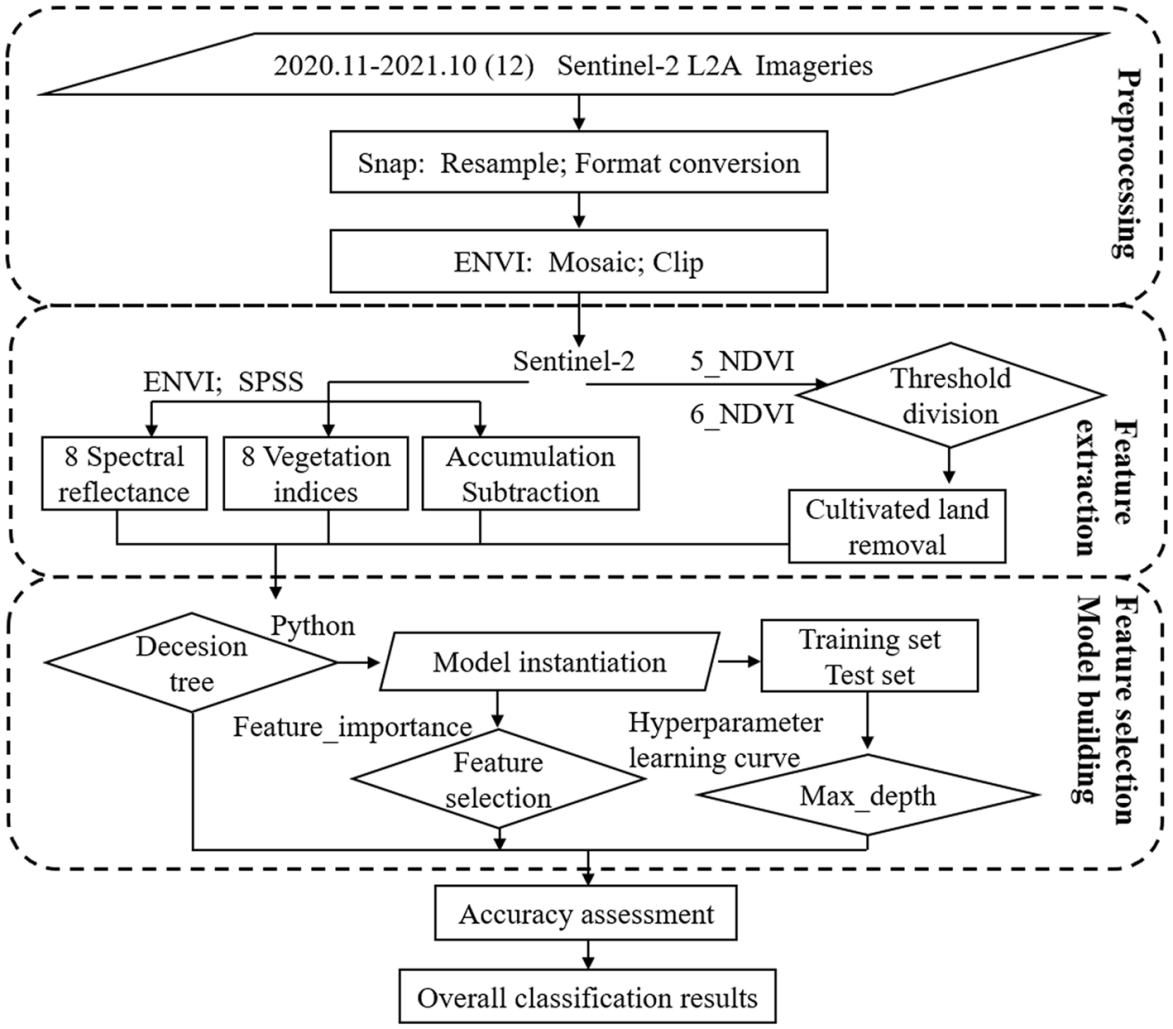
Technical route map. This figure shows the main methods and processes in this study.

This study was based on the fruit tree planting area of the Dasha River Basin, combined with field surveys and satellite remote sensing imageries for spectral data acquisition. Eight vegetation indices were constructed separately from spectral data, the vegetation indices change trend under different treatments was analyzed, and the machine learning feature screening technology was combined to determine the optimal vegetation indices. At the same time, the large NDVI difference between the field crop planting area and the non-field crop planting area in the imageries of May and June was used to eliminate the crop planting area, and the raster imagery part of the maize, wheat, rice, and garlic planting areas was eliminated. The specific technical route is shown in the Fig. 3.

## Results and analysis

### Cultivated land and forest land division

**Figure 4:**
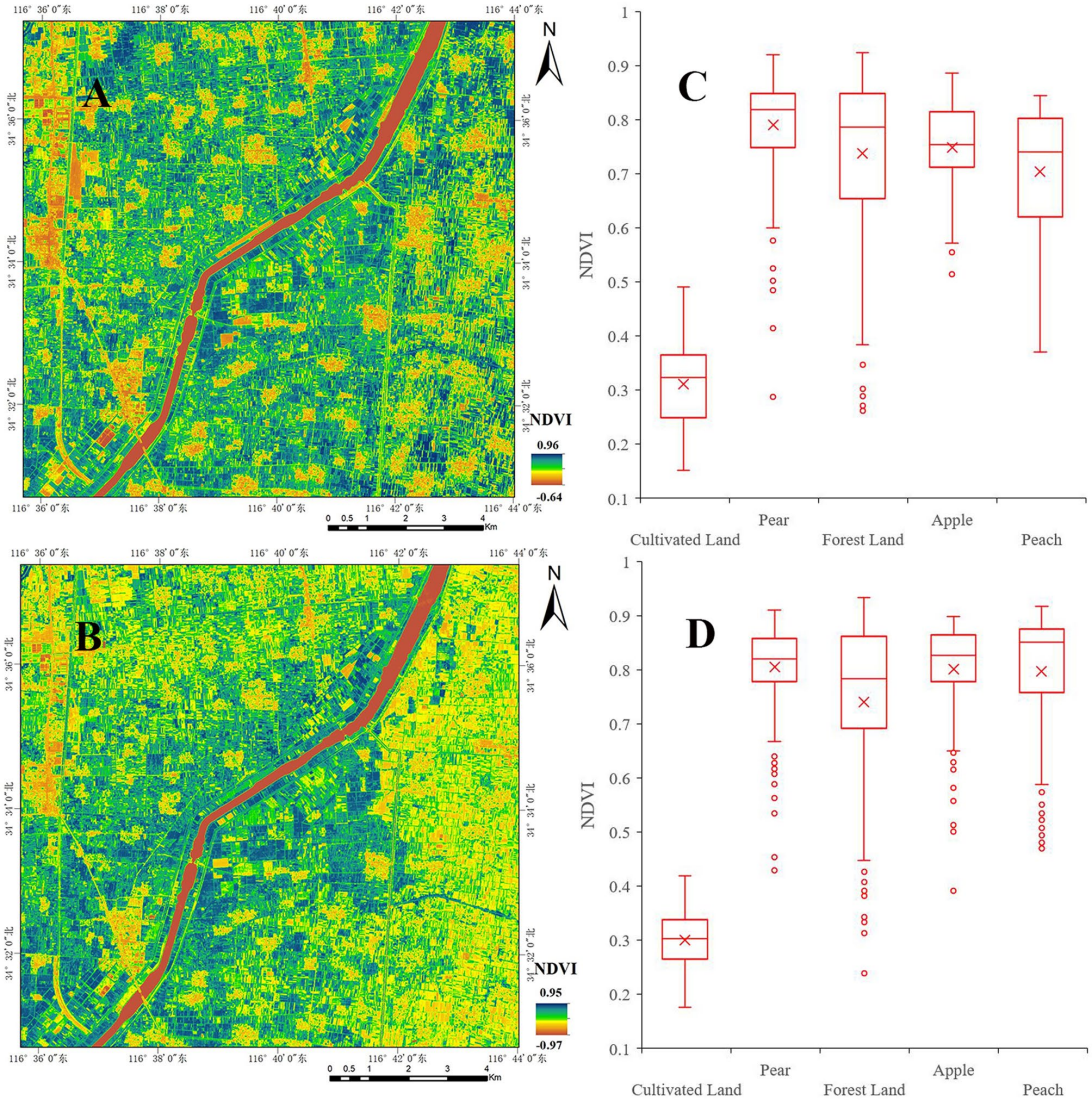
Comparison of NDVI of ground vegetation in May and June. The two figures A and B on the left are the visualization results of the NDVI value in May and June in the study area. The two figures C and D on the right are the NDVI value intervals between the features in May and June in the study area. Map created in ENVI5.3 and ArcMap 10.7 (ESRI Co., USA (https://envi.geoscene.cn/)/(https://www.esri.com/software/arcgis/arcgis-for-desktop)). The satellite imagery data source: Sentinel-2 (https://scihub.copernicus.eu/dhus/#/home).

The planting structure of the Dasha River Basin is relatively complex. Conventional field crops have a great impact on the extraction and classification of fruit tree planting areas. Therefore, the prerequisite for the detailed classification of fruit trees is to effectively remove the cultivated land area. Combining the vegetation growth cycle in this area (Fig. 2), it could be found that the period between May and June was the period of planting and harvesting of multiple crops, and this stage was the growth stage of fruit trees. Therefore, based on this situation, the differences in NDVI between cultivated land and orchards in May and June were analyzed. As shown in Fig. 4, starting from May, the NDVI value of some cultivated land areas was much smaller than the NDVI value of the orchard area, the NDVI value of the cultivated land area was mainly maintained between 0.24–0.36, and the NDVI value of the orchard area was generally higher than 0.6. By June, the difference was even more remarkable. Therefore, based on the huge NDVI difference, the cultivated land area could be eliminated well.

### Feature selection

**Figure 5:**
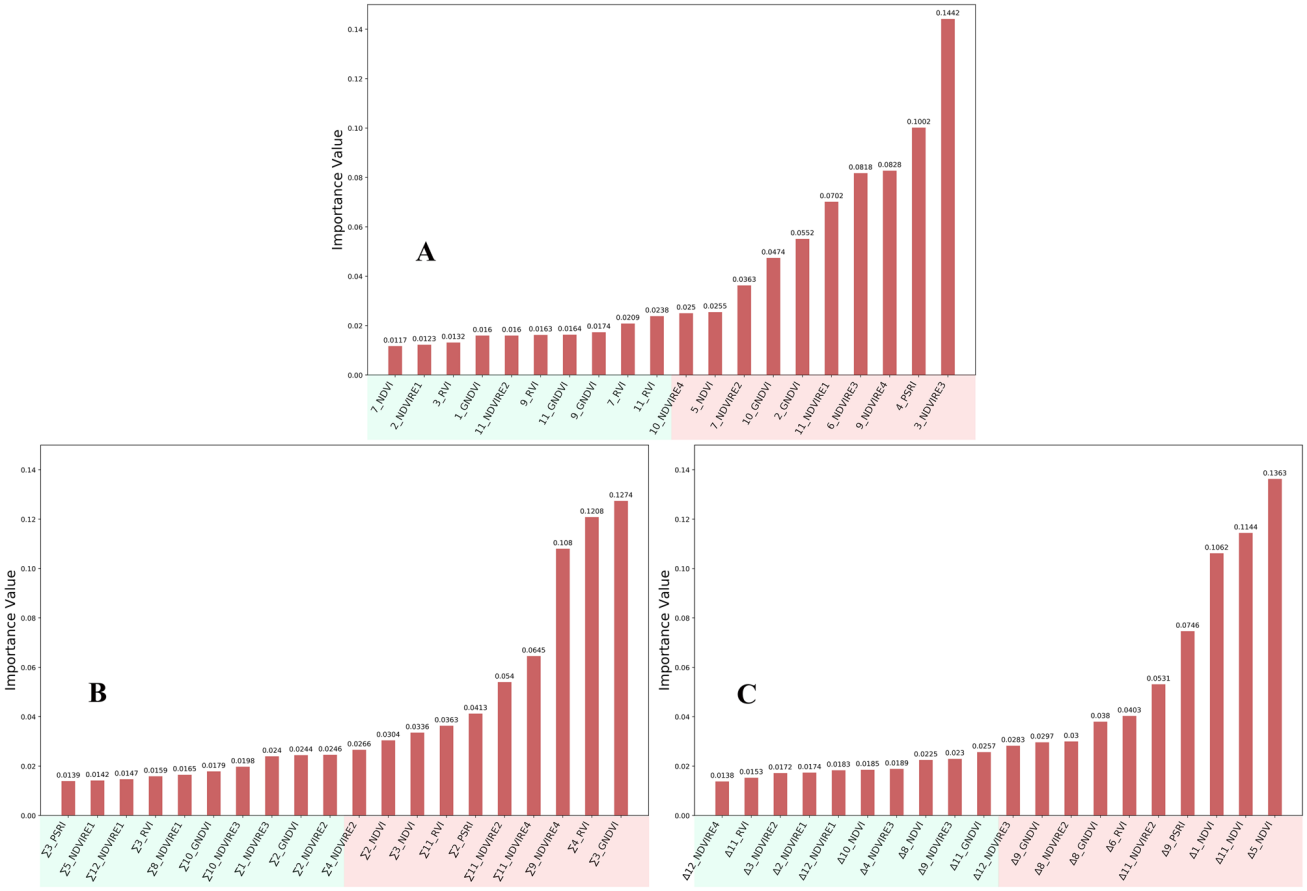
Importance scores of vegetation indices under the three treatments.(** A**,** B**, and** C**) are the scores of SVIs, ∑VIs, and ∆VIs, respectively.

The selection of input features in the decision tree model is particularly important. In this study, a very important attribute of the decision tree, Feature_importances_, could be used to determine the importance of features. The importance of each feature was different, the greater the contribution to the decision tree, the greater the feature importance value. In the case of ensuring the accuracy of the decision tree model and reducing the complexity as much as possible, the top 20 features of the importance value were selected and inputted to the decision tree model. As shown in Fig. 5, the vegetation indices under the three treatments were analyzed to obtain the feature importance ranking. In the SVIs selection, the top 10 features were 3_NDVIre3, 4_PSRI, 9_NDVIre4, 6_NDVIre3, 11_NDVIre1, 2_GNDVI, 10_GNDVI, 7_NDVIre2, 5_NDVI, and 10_NDVIre4; in the ∑VIs selection, the top 10 features were ∑3_GNDVI, ∑4_RVI, ∑9_NDVIre4, ∑11_NDVIre4, ∑11_NDVIre2, ∑2_PSRI, ∑11_RVI, ∑3_NDVI, ∑2_NDVI, and ∑4_NDVIre2; in the ∆VIs selection, the top 10 The characteristics were ∆5_NDVI, ∆11_NDVI, ∆1_NDVI, ∆9_PSRI, ∆11_NDVIre2, ∆6_RVI, ∆8_GNDVI, ∆8_NDVIre2, ∆9_GNDVI, and ∆12_NDVIre3. The importance score of each feature was shown in the figure. The number before the vegetation index represented the month, that was, the vegetation index calculated on the imagery of that month.

### Decision tree depth selection

**Figure 6:**
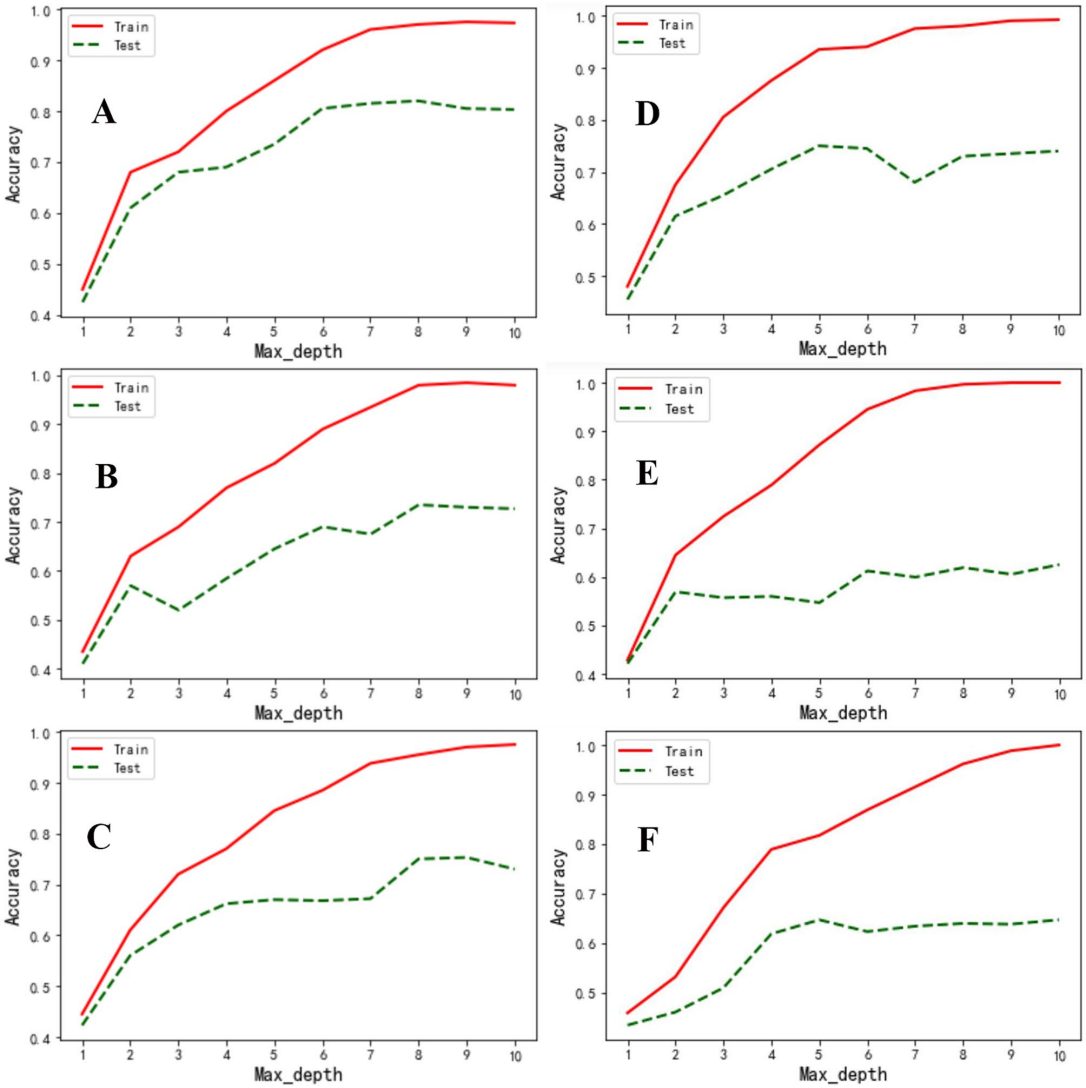
Hyperparameter learning curve of vegetation indices under the three treatments. A, B, and C are the hyperparameter learning curve of the top 20 features of SVIs, ∑VIs, and ∆VIs, respectively. D, E, and F are the hyperparameter learning curves of SVIs, ∑VIs, and ∆VIs without feature screening, respectively.

Without restrictions, the decision tree will grow until the indicator of impurity is the best, or there are no more features available. Such decision trees tend to overfit, i.e., they perform well on the training set, but perform poorly on the test set. The sample data collected in the research cannot be completely consistent with the overall situation. Therefore, when the decision tree has too excellent interpretability for the training data, the rules it finds must include the noise in the training samples and cause insufficient fit of the unknown data. In order to make the decision tree have better generalization, the correct pruning strategy is the core of optimizing the decision tree algorithm. Among them, Max_depth is the most widely used pruning parameter, which can be judged in conjunction with the hyperparameters learning curve. The study used the decision tree model combined with the Matplotlib.pyplot module in Python to draw the learning curve. As shown in the Fig. 6, A, B, and C are the hyperparameter learning curves with SVIs, ∑VIs and ΔVIs as input variables after feature screening, respectively. D, E, and F are the hyperparameter learning curves of SVIs, ∑VIs, and ∆VIs as input variables without feature filtering, respectively. Through comparison, it could be found that when all the features are used as input variables, the phenomenon of model overfitting is aggravated on the features under the three treatments. In SVIs, the test set achieved an excellent result when the Max_depth parameter value reached 6, the accuracy rate exceeded 80%, the generalization ability of the model was the best, and it performed well on the training set and the test set. The ∑VIs and ∆VIs both reached the best results in the test set when the Max_depth parameter value reached 8. It is obvious that the performance of the ∑VIs and ∆VIs values on the test set was worse than the SVIs, and the difference between the training set and the test set was large. After the Max_depth parameter value exceeded 5, there was a certain over-fitting situation.

### Construction of decision tree model

After the input parameters of the model were determined based on the above operation, the decision tree model was constructed, and the final model evaluation was obtained through the Score interface, which the evaluation indicator used the Accuracy coefficient. The scores of the decision tree models of the vegetation indices under the three treatments on the training set and the test set are shown in the Table 4. The results have shown that the SVIs had the best effect on constructing the decision tree model, and its training set and test set scores were 0.8936 and 0.8355, respectively; followed by the ∆VIs, with the test set result reaching 0.7940; The ∑VIs had the worst effect, and the gap between the training set and the test set was the largest. Therefore, the SVIs was selected as the variable input of the decision tree model.

**Table 4:** The performance of the training set and test set of the vegetation indices under the three treatments.

Treatments	Accuracy of Training set	Accuracy of Test set
SVIs	0.8936	0.8355
∑VIs	0.9153	0.7611
∆VIs	0.8887	0.7940

**Figure 7:**
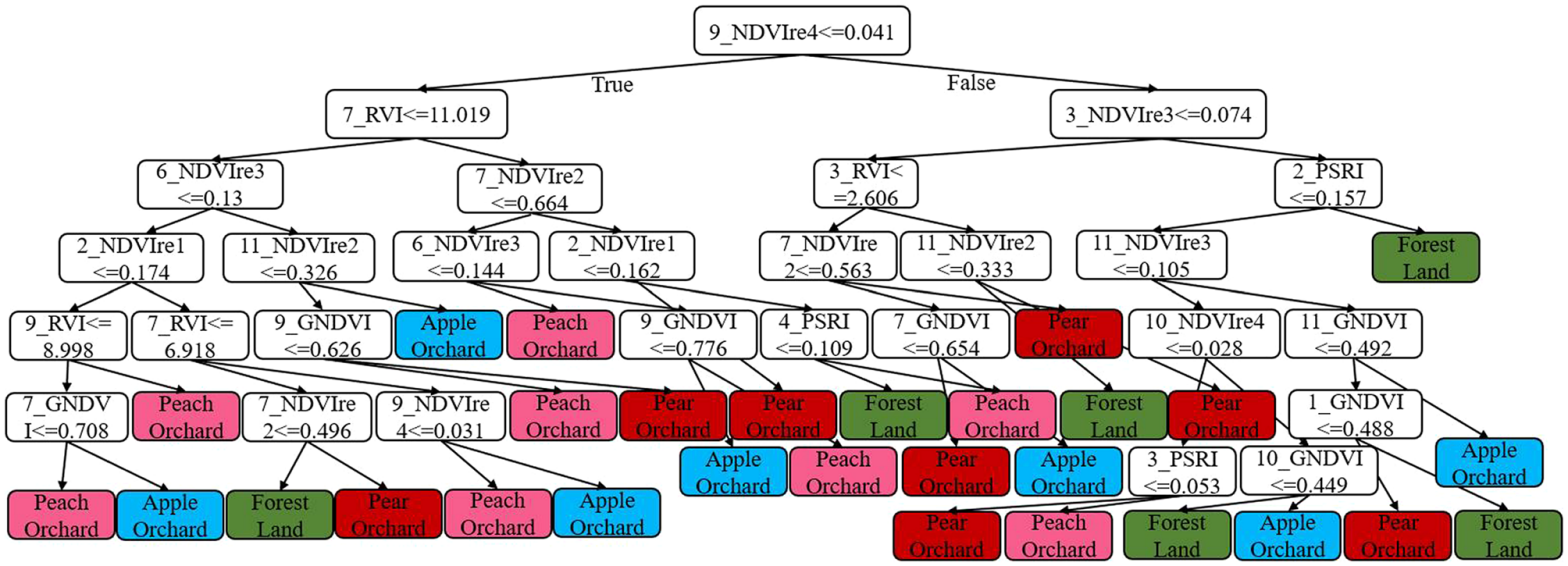
Decision tree model. This figure is a visualization result achieved after the construction of a decision tree model based on SVIs. The depth of the decision tree model is 6, and the final tags are reflected in each branch.

**Figure 8:**
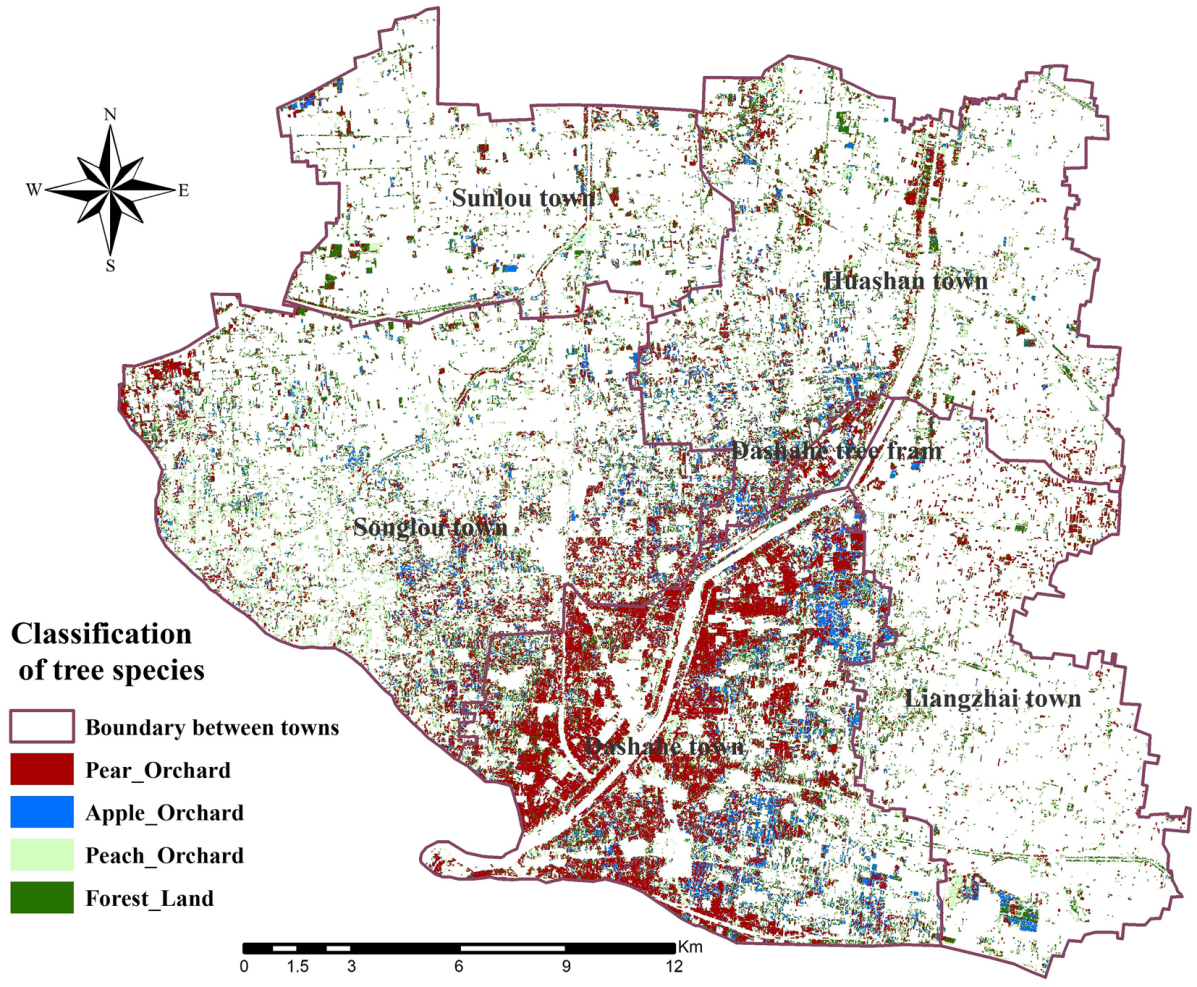
Results of remote sensing classification of fruit tree planting area. The figure shows the spatial distribution of pear orchards, apple orchards, peach orchards, and forest land. Map created in ENVI5.3 and ArcMap 10.7 (ESRI Co., USA (https://envi.geoscene.cn/)/(https://www.esri.com/software/arcgis/arcgis-for-desktop)). The satellite imagery data ). source: Sentinel-2 (https://scihub.copernicus.eu/dhus/#/home.

The visualization of the decision tree model used the Graphviz module and the Tree.export_graphviz class, as shown in Fig. 7. Based on the decision tree model under the machine learning technology, the Sentinel-2 imagery of each month was selected, and the ENVI5.3 software was used to perform band calculations to generate the single-band raster imagery of the vegetation index of the model input. The above decision tree model results were established and executed on ENVI5.3 software respectively, combined with ArcGIS 10.7 software to realize the drawing of classification thematic maps, and the results shown in Fig. 8 were obtained. The results have shown that the main planting areas of fruit trees were basically around the two banks of the Dasha River, and most of them were continuous planting. The plots of fruit tree planting in the east and northwest were relatively fragmented. Among them, pear trees were mostly located on both sides of the Dasha River, apple trees were mainly distributed in Dashahe Town and Liangzhai Town, peach trees were scattered, and relatively more in Huashan Town. The results of the spatial distribution monitoring were basically consistent with the field surveys.

### Classification accuracy verification

In order to objectively and quantitatively evaluate the effect of the fruit tree classification of the decision tree model, this study used the confusion matrix method to evaluate the accuracy. Random verification points were created in the study area, a total of 600 random points, combined with high-resolution imageries and field surveys, each random point was visually interpreted, and compared with the remote sensing classification results, and a confusion matrix was made. Then the user accuracy, mapping accuracy and Kappa coefficient were calculated separately (Table 5). The user accuracy of pear orchard, apple orchard, peach orchard, and forest land were 83.33%, 82.71%, 78.57%, and 80.64%, respectively. The mapping accuracy were 82.78%, 81.35%, 77.46%, and 79.71%, and the Kappa coefficient was 0.8140. The results have shown that the classification results of peach trees were relatively poor, but the overall error rate and miss rate were low. The two accuracy verification results based on the scores of the test set and the training set and the confusion matrix verified that the remote sensing classification model of fruit tree planting areas in this study had high recognition accuracy, and could realize the remote sensing monitoring of the spatial distribution of fruit trees in the area.

**Table 5:** Classification accuracy results.

Classes	User accuracy	Mapping accuracy	Kappa coefficient
Pear orchard	83.33%	82.78%	0.8140
Apple orchard	82.71%	81.35%	
Peach orchard	78.57%	77.46%	
Forest land	80.64%	79.71%	

## Discussion

Currently, the acquisition of plant information at the remote sensing level has mainly based on optical satellite data. In optical remote sensing research, the distribution of crops is usually determined by analyzing the changes in the spectral reflectance and vegetation index of the current image. The limitation is that in the same period, there is a phenomenon of "same spectrum with different objects" between fruit trees and other plants. Therefore, simply based on the single-phase imagery and spectral characteristics cannot distinguish various objects^40^. In addition, the remote sensing imageries used in the plant classification were mainly originated via MODIS, NOAA/AVHRR, etc.^41,42^. These imageries were difficult to apply to high-precision fruit tree remote sensing classification in small areas because of low spatial resolution. On the other hand, the high-resolution imageries such as Quickbird, SPOT, IKONOS were costly^43^. The medium-resolution TM imageries had revisiting periods of 16 days, making it difficult to obtain high-quality data in time. This limited continuous fruit tree monitoring and made it inappropriate to classify the species of tree^44^. As a rising star, the ESA sentinel series of data has been continuously improved and was provided free of charge to users. This has created a convenient data platform for remote sensing classification of regional crop’s distribution. The experimental area of the present research has been located in yellow river flooded alluvial plain in northern Jiangsu Province. The cultivated land and orchard have been fragmented and as a result the planting structure was complex. The time resolution of the selected Sentinel-2 imagery was 4 d, and the scanning width of the single scene imagery was 290 km. These characteristics could meet the classification demands for the actual regional type of fruit tree.

In this study, the Sentinel-2 time series imageries covering the whole growth period of fruit trees were used to explore its influence on the classification of fruit trees by constructing SVIs, ∑VIs, and ∆VIs, and the vegetation indices of the relevant period were calculated as the input feature. Combining the machine learning technology under the Python framework to construct the best decision tree model, it achieved a higher-precision effect of distinguishing fruit tree types, effectively extracted different fruit tree planting areas, with a higher overall accuracy. The red edge band is the region between 680 and 750 nm and is considered the most significant sign of green vegetation. Because it has the point where the reflectance rises the fastest, this point is also known as the maximum value of the first derivative of the plant spectrum in this wavelength range^45^. It is obvious that the improved normalized vegetation index based on the red edge band as an input feature has a significant role in the classification model. Either in the feature selection or in the visualization results of the decision tree model, it is apparent that the improved vegetation index constructed by the red edge band has a significant imagery factor for the classification model, which is consistent with the research of Luo et al.^46^.

In the past research on ground object classification based on remote sensing, the main method was to describe plant growth characteristics, and based on artificial experience, data analysis and threshold division were carried out to draw decision trees^47–49^. For optical remote sensing, the target extraction is performed by analyzing the spectral reflectance of different fruit trees and other plants and constructing a vegetation index. The limitation is that the canopy spectral information changes slightly, and the complex farmland environment also increases the difficulty of spectral information extraction and threshold division. Therefore, this type of decision tree model has good readability and simplicity, it is difficult to achieve the most ideal state in classification accuracy. Traditional remote sensing extraction methods have been difficult to meet the current needs of agricultural high-precision extraction. With the development of machine learning technology, the application of feature engineering technology has sprung up, and effective feature screening can play an important role. At the same time, the hyperparameters learning curve is of great significance in exploring the application of the maximum potential of the model. Hence, it is necessary to introduce machine learning ideas to improve the accuracy of decision tree classification models in scientific research or production.

Although the overall accuracy of the detailed classification of fruit trees in this study was relatively high, there were also cases where the classification accuracy of some categories was relatively poor. Among them, the classification results of pear trees and apple trees were better than peach trees. The reason was that the local peach tree planting structure was relatively complicated, and there were a large number of early and late varieties. Apples were mainly late-maturing Fuji varieties, and pears were mainly early-maturing varieties. In consequence, the classification model had a better effect on fruit tree types with a more consistent growth cycle, but it was naturally less effective in a group with a more chaotic growth cycle. At the same time, forest land had a great influence on the classification results of fruit trees. Due to the same tree types, the annual growth cycle of forest land and fruit trees overlapped more. There were many types of forest land trees, and the corresponding vegetation indices ranges in the same period were quite different. Therefore, in view of the errors and misclassifications in the process of fruit tree classification, the proportion of forest land was relatively large.

In the study, it was found that the more variables used to construct the decision tree model is not the better. Models built from variables without feature selection tend to perform equally well on the training set, but perform poorly on the test set. The redundancy of data and the interference of noise will lead to the development of the model in the direction of overfitting, so effective feature screening is very necessary. Regarding the performance of the three vegetation indices in the classification model, it could be found that the SVIs as an input variable has the best effect. Its scores on the training set and test set were 0.8936 and 0.8355, respectively. (Fig. 6). While ∑VIs and ∆VIs are used as input variables, the scores of the models constructed by them on the test set are both less than 0.8. The reason may be that the processing of accumulation and difference between vegetation indices cannot effectively amplify the spectral information differences between target vegetation types, resulting in unsatisfactory classification results.

The planting structure in the study area is complex, and there is a certain phenomenon of "same spectrum with different objects". It is difficult to identify and extract objects using single-phase remote sensing data. Multi-temporal remote sensing data sources can effectively reduce the impact of this phenomenon. In the follow-up research work, the focus will be on combining more types of remote sensing data sources and introducing other features to improve its impact. The present study did not compare the decision tree model with other classification models such as random forest (RF), support vector machine (SVM), etc. The main research content focused on the performance of multi-temporal remote sensing data combined with feature engineering technology under the decision tree model. Other classification algorithms combined with the idea of feature engineering techniques in this study may yield better results and needed further study.

## Conclusion

In the present research, four conventional vegetation indices and four improved vegetation indices based on the red edge band were used to classify fruit tree planting areas. SVIs, ∑VIs, and ∆VIs were analyzed respectively, and selection of features and parameters of decision tree model were carried out by machine learning technology. The result was that the accuracy of the decision tree model constructed by the vegetation index under the three treatments were 0.8936, 0.9153, and 0.8887 on the training set, and the accuracy on the test set were 0.8355, 0.7611, and 0.7940, respectively. The decision tree model based on SVIs could meet the remote sensing classification of fruit trees in a large area and provide effective technical means for remote sensing imagery monitoring of fruit tree planting areas. However, at the same time, it should be considered that there are many rainy weather conditions in the study area, which makes it difficult to obtain optical images. Future work will use more types of remote sensing data sources (such as synthetic aperture radar) and other features (such as texture information) to explore the application of different classification methods.

## Data Availability

The datasets used and/or analysed during the current study available from the corresponding author on reasonable request.
